# Environmental Stressors and Adaptive Mechanisms in Long-Term Care Resident Bedrooms: A Canadian Case Study

**DOI:** 10.1177/19375867251406198

**Published:** 2026-01-06

**Authors:** Alexandra Boissonneault, Terri Peters

**Affiliations:** 1 Department of Architectural Science, 7984 Toronto Metropolitan University, Toronto, Canada

**Keywords:** long-term care facilities, postoccupancy evaluation (POE), resident well-being, environmental stressors, adaptive comfort mechanisms

## Abstract

**Aim:**

The aim of this research was to identify the adaptive comfort mechanisms available in resident bedrooms of a newly built long-term care (LTC) home in Ontario, Canada, and examine how these were used to manage environmental stressors.

**Background:**

LTC facilities house vulnerable populations susceptible to various stressors. While psychosocial stressors are well-documented, physical stressors caused by the indoor environment also significantly impact resident behavior and satisfaction.

**Method:**

This study was nested within a larger mixed-methods postoccupancy evaluation. Methods included building walkthroughs, spot measurements, structured observations, staff and resident interviews, and an analysis of network sensor data extracted from building system reports.

**Results:**

Few adaptive mechanisms were available to residents. Those that were—window blinds and adjustable thermostats—were being actively used to mitigate perceived environmental stressors. While light levels in resident bedrooms met minimum requirements, frequent use of blinds and reliance on artificial lighting diminished the benefits of daylight on health and well-being. Temperature trends indicated frequent fluctuation within the acceptable thresholds and greater sensitivity to heat than cold.

**Conclusions:**

Personal control mechanisms play a critical role in enhancing resident comfort in LTC facilities. Despite meeting regulatory standards for indoor environment quality, blind and thermostat use revealed additional layers of environmental stressors that need to be addressed along with critical opportunities for further research. Limitations in the accessibility and usability of personal control devices for residents with mobility or cognitive impairments highlight significant areas for improvement in LTC design.

In long-term care (LTC) settings, residents are susceptible to a range of stressors, both physical and psychosocial (Angevaare et al., 2020). While psychosocial stressors, such as social isolation, loss of autonomy, and the emotional strain of managing chronic illnesses, and their effects are well documented (e.g., Angevaare et al., 2020, 2022; Blazer, 2025; Lem et al., 2021; Mulla et al., 2025), less attention has been paid in the literature to physical stressors within their indoor environments, including noise, air quality, lighting, temperature, and space constraints (Bluyssen, 2014). These environmental factors are known to affect residents’ health, satisfaction, comfort and well-being (e.g., Ausserhofer et al., 2016; Joseph et al., 2016; Sion et al., 2020; Van Steenwinkel et al., 2017).

In Canada, LTC design and operations are governed by numerous regulations (Daly, 2015), and opportunities for personal control over one's surroundings are limited. However, evolving standards and best practices increasingly emphasize the importance of autonomy and choice among older adults in residential care. This article presents findings from a secondary study nested within a larger mixed-methods, multiphase postoccupancy evaluation (POE) of a newly built LTC home, in Ontario, Canada. The broader POE had as its primary objective exploring resident and staff experiences of interinstitutional relocation from an old familiar LTC home to a new, much larger, more modern facility. The study summarized in this article focused specifically on physical environmental stressors and adaptive mechanisms within resident bedrooms—spaces that are deeply personal and restorative, and central to supporting comfort, control, and dignity in LTC (Bae & Kim, 2024). It sought to answer the following research questions: What adaptive mechanisms were available to residents in the new home and how were these mechanisms used to manage perceived environmental stressors?

As improving the quality of residential elder care remains a global concern, this study aimed to highlight an underexplored aspect of the resident–environment relationship. Its contribution lies not in generalizability, but in identifying critical opportunities for more comprehensive and targeted research on stressors and adaptations among resident as well as other users in LTC.

## Background

### POE and Indoor Environment Quality

POE is an established approach in both basic and applied building research to systematically collect feedback on buildings in use for the purposes of exploration, investigation and/or diagnosis (Preiser et al., 1988). It is commonly used to assess indoor environment quality (IEQ) and its effects on occupant health and comfort (Li et al., 2018). These studies are most often conducted in office environments, where occupant well-being is closely tied to productivity and profit (Boissonneault & Peters, 2023). POEs have also been used to explore the tradeoffs between IEQ, occupant comfort and building performance (e.g., Laskari et al., 2022; Vivian et al., 2023; Xiaodong, 2018). Few studies examine the bidirectional relationship between individuals and their physical surroundings—that is, how people interact with and adapt their environments to meet personal needs and preferences (Vischer, 2008). This gap is evident in LTC research. In LTC settings, residents often face physical or cognitive challenges that constrain their ability to modify or respond to their environment, increasing vulnerability to discomfort and diminished well-being. Despite these constraints, autonomy and personalization are highly valued by residents (Van Hecke et al., 2019; van Loon et al., 2024; Van Steenwinkel et al., 2017). To date, POEs on IEQ in LTC have focused largely on resident satisfaction, however (e.g., Huang et al., 2013; Mu & Kang, 2022; Zhan et al., 2021), with limited attention to adaptive behaviors or environmental control mechanisms. One exception is a study by Tartarini et al. (2018) which investigated thermal perceptions, preferences, and adaptive behaviors of nursing home residents using field measurements and surveys. While it contributed insights into temperature tolerance and the limitations of standard thermal comfort models (e.g., PMV) for older adults, it did not examine the role or impact of new features like adjustable thermostats in contemporary LTC design. Research on this topic is needed to inform more responsive, inclusive, and sustainable design strategies.

### Stressors and Adaptations

Adaptations are a defining aspect of user-building interactions to improve comfort or fit (Willems et al., 2022). They typically occur in response to actual or perceived environmental stressors (Zeisel, 1981). These stressors, such as temperature fluctuations, poor lighting, noise, crowding, and inadequate air quality, can be particularly pronounced in new or underperforming indoor environments. Older adults in LTC are especially vulnerable to such stressors due to their physiological and cognitive frailty (Cubukcuoglu et al., 2023). As Bluyssen (2014) outlines, exposure to environmental stressors—acute or chronic—can trigger physiological responses and contribute to a range of health issues, including cardiovascular disease, respiratory illness, obesity, diabetes, and depression. Additionally, poor access to daylight during the day has been linked to disrupted sleep (Goudriaan et al., 2021). The significance of indoor environmental quality in LTC settings gained heightened public attention during the COVID-19 pandemic, when poor air quality and ventilation were identified as key factors in infection rates and severity in Canadian facilities (Marrocco et al., 2021). Rather than examining the effects of stress factors, this study focuses on the limited adaptive mechanisms available to residents in this highly regulated sector, and the insights that can be drawn from their use.

### The LTC Context and Case Study Building

**Figure 1.**
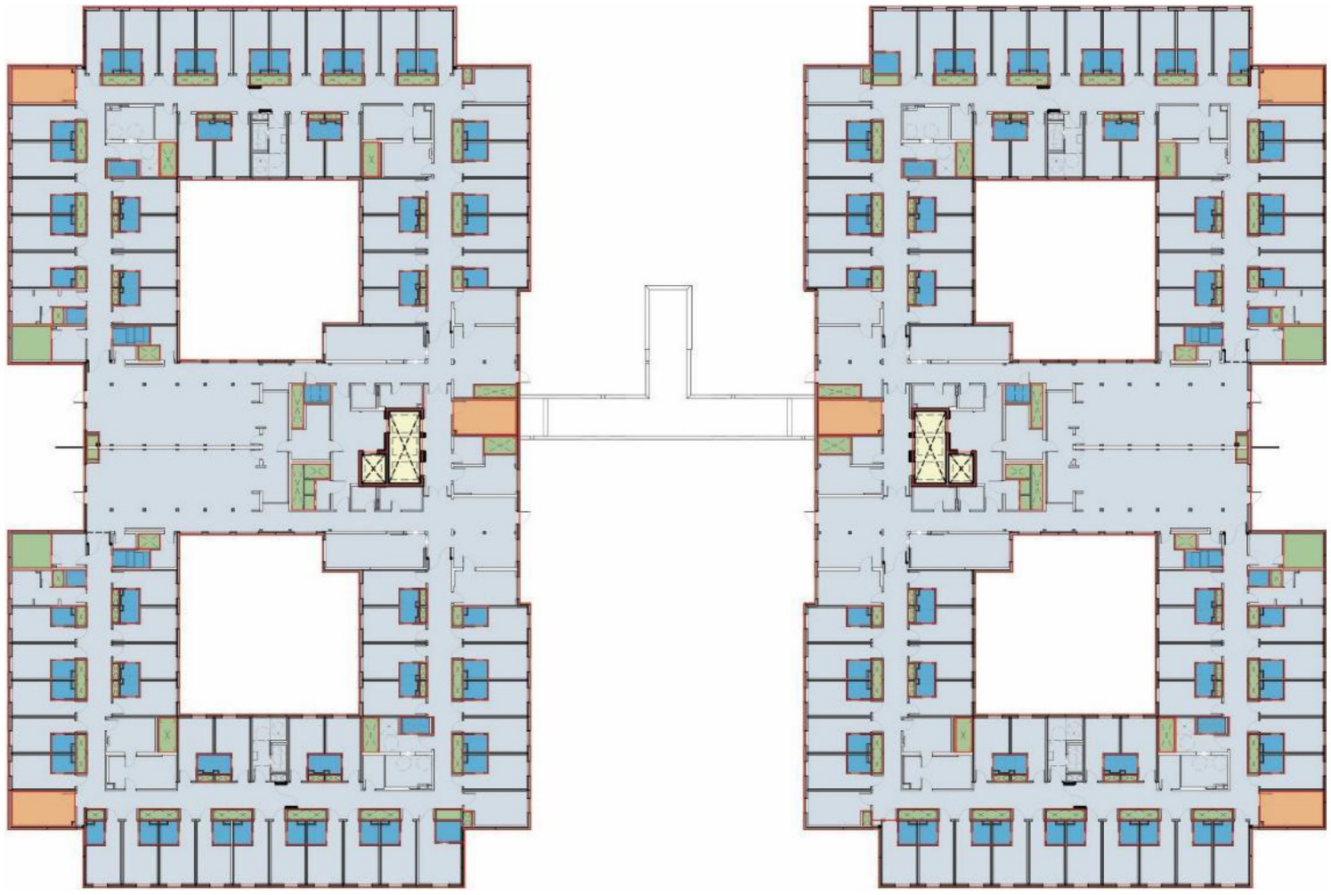
Typical floorplan of the case study building showing the distribution of resident bedrooms around a series of courtyards.

**Figure 2.**
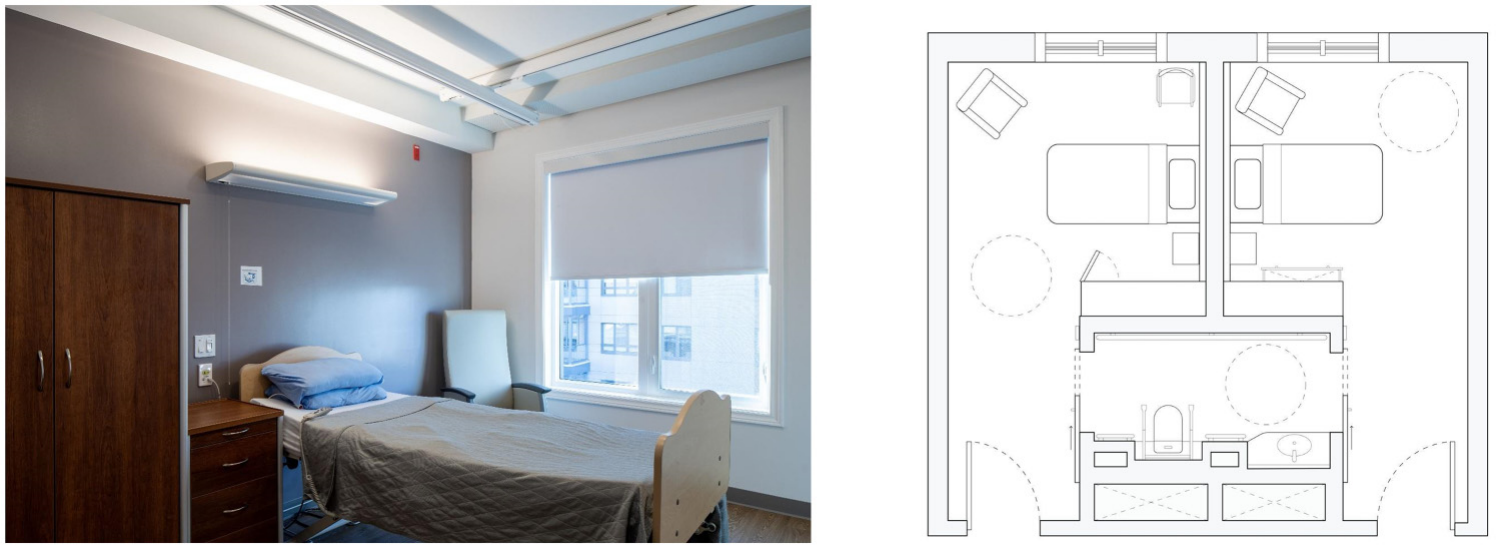
Image of typical bedroom overlooking the courtyard and corresponding plan, semiprivate with shared ensuite.

In Ontario, every facility must comply with the Ministry's current LTC Home Design Manual (2015). The LTC Home Design Manual, 2015 sets minimum standards for resident, staff and public spaces, including IEQ and the latitude of calibration, to ensure parity across providers (Daly, 2015). The case study building, located in central Ontario, is a representative example of ongoing government efforts to redevelop and modernize ageing LTC infrastructure across the province as well as meet ever-growing demands for beds in certain regions. It both replaced an outdated LTC home and more than doubled its capacity, accommodating 632 beds across two adjoining towers. Illustrated in Figure 1, beds are divided into 32-bed home areas. In each home area, a mix of private and semiprivate rooms line a looping double-loaded corridor that encloses a central courtyard. A typical bedroom plan is illustrated in Figure 2. At the time of the POE, 90% of the beds were occupied. As with other new facilities, the case study building placed greater emphasis on IEQ for the purpose of occupant health, comfort and quality of life. Creating opportunities for personal control, particularly in spaces like the bedroom and shower room, was a key design driver with the intent to allow occupants to calibrate their space per their individual needs and preferences. With growing investment in new LTC infrastructure, this article identifies a research gap and a need for research that examines how residents exercise agency through personal control devices, and how environmental stressors and their management affect user experience, building operations, and energy performance.

## Methodology

This study was nested in the design of the larger POE, building on preliminary data analysis and emerging themes. The larger POE was designed as a mixed-methods case study with a convergent design, allowing for quantitative and qualitative data to be mutually informing (Creswell & Plano Clark, 2006). Data collection occurred over a 10-week period, 8 months after building occupancy. While POEs are typically conducted a minimum of 1–2 years after operationalization to avoid premature feedback (Preiser et al., 1988), the expressed scope of this evaluation was the period of acclimatization postrelocation and the physical and psychosocial stressors impacting occupant perceptions of and interactions with their changed environment.

Qualitative methods, human participation and recruitment were previously published in detail in Boissonneault et al. (2025) along with a subset of findings. Interpretive Description (ID) was used as the qualitative framework as it is suitable for exploring complex experiential questions in applied settings (Thorne et al., 2016). A total of 19 participants were interviewed—14 staff and 5 residents. Staff were selected through purposive and snowball sampling to ensure diversity in roles and experiences. Resident recruitment was facilitated by a member of the care team who acted as a gatekeeper, using a cognitive ability assessment model to identify eligible and willing participants. Given the overall objective of the POE, only staff and residents who had relocated from the old home were eligible to participate. All interviews were conducted in person with one exception via Zoom, audio-recorded (except for three who decline this option), and transcribed using AI-assisted software, then manually cleaned and coded by the lead author to identify preliminary patterns and themes, which were refined through constant comparative analysis and collaborative interpretation with the coauthors. Per Thompson Burdine et al. (2021), rigor was ensured through member checking, expert scrutiny, detailed memoing, and a comprehensive audit trail documenting all analytical processes. Ethics approval was obtained from the Toronto Metropolitan University Research Ethics Board (No. 2024-074). Verbal consent was obtained from all participants prior to participation. All participation was voluntary and confidential.

Quantitative methods involved walkthroughs to verify the functioning of the new building systems and document the technical attributes of regularly occupied spaces, including the resident bedroom. In tandem with walkthroughs, spot measurements were taken of temperature, humidity, and illuminance using handheld devices in a sampling of these spaces across multiple floors and orientations to measure actual indoor environment conditions. Walkthroughs and physical measurements are typical POE methods, allowing the researcher to understand and record the physical conditions of the building and contextualize and compare user feedback (Wener et al., 2016). Measurements were tabulated in a spreadsheet and compared against the LTC Design Manual standards for temperature and illuminance to identify the presence of physical environmental stressors.

Quantitative walkthroughs identified two new personal control mechanisms available to residents in their bedroom: adjustable thermostats and horizontal roller blinds. Emerging themes related to environmental stressors and personal control during the initial data analysis informed the development of this study's research question and incited additional targeted data collection with which to converge and compare results. This data collection involved structured observations of what Zeisel (1981) characterize as “adaptive traces” in resident bedrooms, including evidence of the use of personal control mechanisms in response to actual or perceived stressors. Photographs and field notes were used to document observations. It also involved the collection and analysis of network sensor data from monthly building system reports generated by the building automation system on temperature values across a random sample resident bedroom (*n* = 82). Data sets were cleaned, and descriptive statistics were generated to analyze sensor data points, with a focus on average and absolute minimum, maximum and mean values as well as standard deviations for each parameter calculated over 24-hr cycles to identify central tendencies and variability within the dataset. Data points were then plotted using a Python-based data visualization tools in Matplotlib to visualize the distribution, trends and outliers relative to ministry thresholds. Due to the constraints of the POE and limited control over the study context, further data collection and more in-depth analysis were not feasible. Recognizing these limitations, the study aimed not to produce generalizable findings, but to identify patterns and trends that could prompt future research on, among other things, mediating and moderating factors among users (Table 1).

**Table 1.** Indoor Environmental Quality Measurement Specifications.

IEQ Parameter	Measurement Device	Measured Indicator/Margin of Error	Measurement Location	Measurement Intervals
Visual comfort	Dr. meter Lx 1330B	Illuminance (lux) at horizontal plane, 914 mm from floor /±3% ± 10 digits	Resident bed, 24 in from floor	One-time measure
Thermal comfort	Johnson Control N8000 Series Network Sensor	Air temperature (°F) / ± 0.5°C (±0.9°F)	Resident bedrooms, interior wall	10-min intervals, continuous over five typical days
	Aranet4	Air temperature (°C) / ± 0.3 °C (±0.5 °F)	Resident bed, 24 in from floor	10 min, continuous
		Relative humidity (%) / ± 3%	Resident bed, 24 in from floor	10 min, continuous

Iterative analysis of the different data sources and data sets was then undertaken in which the authors moved between the study-specific and broader POE findings to generate meta-inferences (Greene, 2008). Joint display tables were used to facilitate integration and interpretation of numerical data, visualizations, photographs, field notes, and representative quotes.

## Results

The technical attributes related to visual and thermal comfort in resident bedrooms are summarized in Table 2.

**Table 2.** Technical Attributes Related to Visual and Thermal Comfort in Resident Bedroom*s*.

Attribute	Descriptions
Ceiling fixture type and shape	Recessed pot light 100 mm–150 mm (4”–6”), circular
Ceiling height	3200 mm (10’-6”)
Ceiling light lens type	Linear spread lens
Level of ceiling light control	Individual control in bedrooms. On–off, continuous dimming. Occupancy sensors in washrooms.
Task lights	Fixed, 1 near the headboard of the bedrooms.
Daylight effectiveness	1524 mm (60”) × 1803 mm (71”) bedroom window size. Side lit, single orientation windows.
Window controls	Roll down interior opaque shades, manual operation.
Size of zone in core (No. of people/thermostat)	Thermostat in each private or semiprivate room.
Core system type	Rooftop air-handling units with heat recovery units (HRUs) and variable air volume fans in common areas; HRU at bedrooms and washrooms.
Level of thermostat control	Adjustable thermostat with setpoint and status.
Seasonal switchover	Each zone continuous control.
Humidity control and ventilation	Central humidity sensing; no humidification or dehumidification for individual rooms; HRU's with supply and exhaust fans, filters, and gas humidifiers are provided, run continuously to provide tempered air to each bedroom.
Dedicated exhausts	None.
Window quality	Individual internal shades.
Window controls	Operable windows in bedrooms to 4” max. Opaque vertical blinds.

### Lighting Conditions and Adaptive Behaviors

Each bedroom had a window with roller blinds that can be adjusted to provide daylight and views, and a light switch for overhead electric lighting that can be turned off or on. The LTC Home Design Manual requires continuous illumination at 322.92 lux in resident areas of the home including resident bedrooms. This can be delivered via daylight or electric lighting or a mix of both. The illuminance levels measured in the sampling of resident bedrooms was aligned with the criteria outlined in the LTC Home Design Manual. Regardless of whether the blinds were drawn, bedrooms met the required standards for visibility when electric lighting was on.

**Figure 3.**
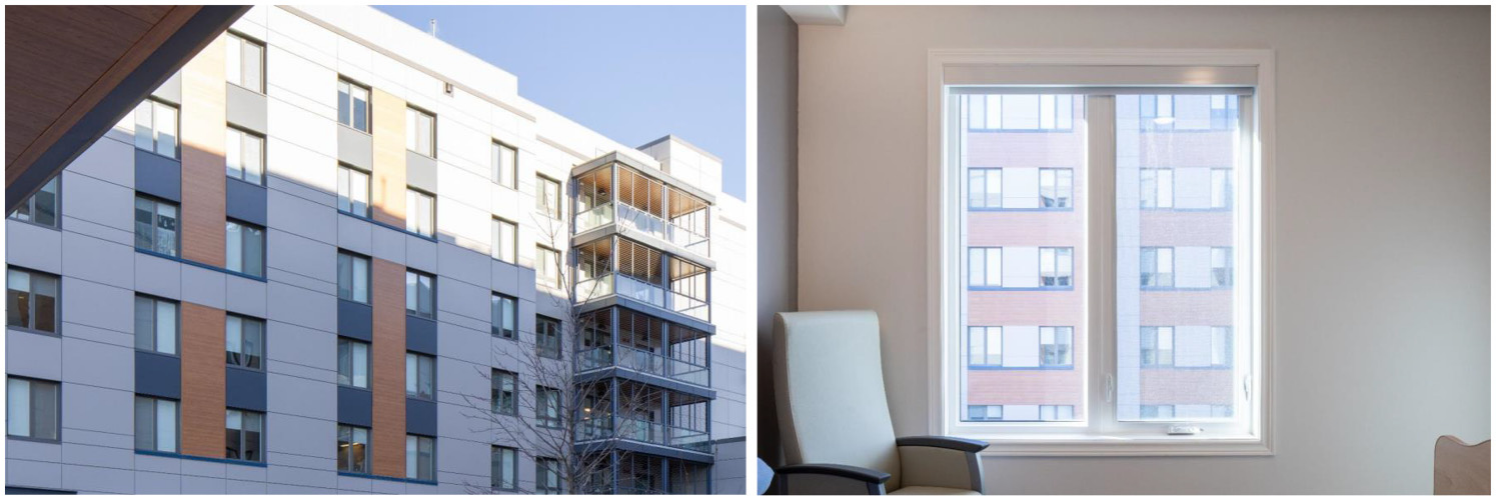
Blinds drawn in courtyard-facing rooms, as observed from both the courtyard and resident bedroom.

Qualitative accounts identified no issues or potential stressors with regard to electric lighting across the facility in terms of general or task lighting. However, participants voiced recurring concerns around heat gain often tied to a fear that medical equipment, such as oxygen tanks, might overheat. Illustrated in Figure 3, they also indicated residents overlooking the courtyard preferred to keep their blinds closed for reasons of privacy such that when blinds were open, “Residents can look into each other[s] windows and they know that people can see them. But let's say it's in the middle of the day – do I necessarily want to have my blinds pulled when I'm doing something?” (C12). Participants felt the problem was exacerbated by the lower sill heights designed to improve views from the bed.

Reinforcing qualitative data, observation of adaptive traces found that blinds were frequently drawn in courtyard-facing bedrooms. Irrespective of orientation, residents were also observed to sit away from their windows when not in bed. These behaviors limited resident access to daylight and views, which was a design intent.

### Thermal Conditions and Thermostat Use

**Figure 4.**
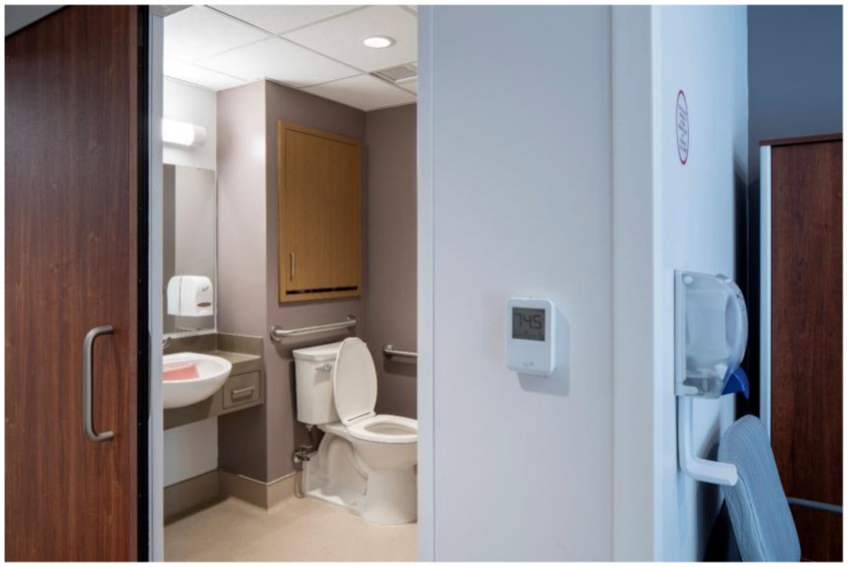
Thermostat with built-in sensor on interior bedroom wall at typical mounting height of 1.2 m (3.28 feet).

Shown in Figure 4, thermostats were installed on interior walls of resident bedrooms, consistent with standard practice, at a typical mounting height of 1.2 m (3.28 feet). Notwithstanding some ongoing commissioning, the systems were found to be performing as expected. Spot measurements of the sample bedrooms recorded temperatures within the Ministry approved threshold of 22–25.9°C (71.6–78.62°F). No actual physical stressors were readily apparent in the indoor environment.

**Figure 5.**
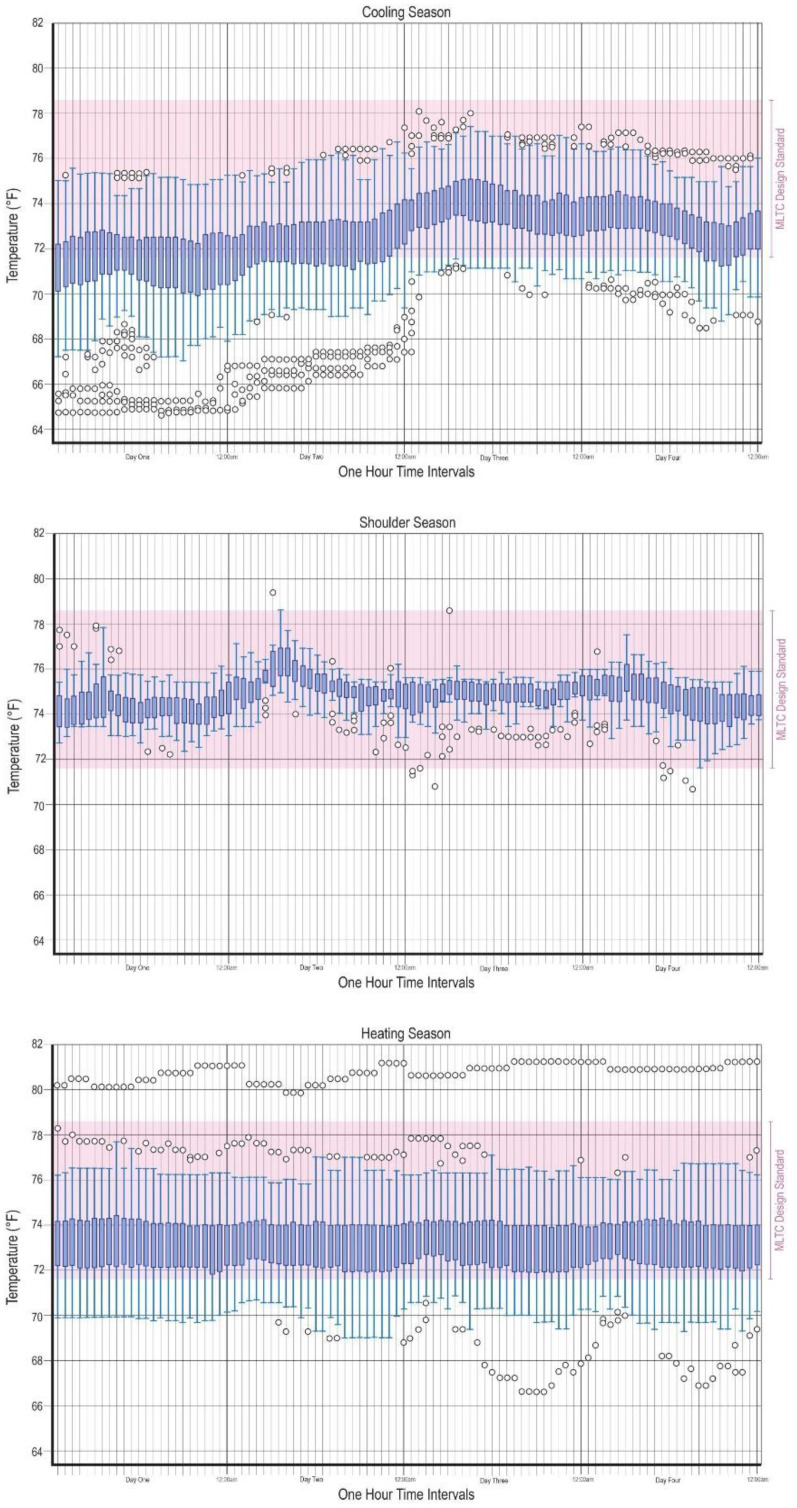
Resident room temperatures during heating, cooling, and shoulder season plotted at 1-hour intervals over 24 hr cycles.

Qualitative findings indicated however that temperature was a recurring complaint among occupants. Most interview participants perceived thermostats in resident bedrooms as a positive mitigating feature and noted they were being used regularly. Some, however, felt that changing the set point had a limited effect on room temperature and that thermal discomfort often persisted. From a facilities perspective, maintaining individual thermostats and ensuring rooms were within the mandated thresholds required ongoing monitoring and management. When bedrooms set points and/or temperatures exceeded the given thresholds and/or an irregularity was identified, a dedicated technician was deployed to address the issue.

Temperature trend reports from the building automated system suggest thermostats in resident bedrooms were actively being used and therefore thermal discomfort was a perceived environmental stressor in resident bedrooms. As shown in Figure 5, overall, temperature trends followed a typical pattern reflective of occupancy, external weather and equipment usage, gradually increasing in the morning hours, reaching peak values in the midday, declining through the afternoon and stabilizing into the evening and overnight. Some bedroom thermostat data showed relatively stable temperatures with minor fluctuations. Others exhibit more significant variations. More outliers were found in the heating season suggesting possible sensor errors. They may also be indicative of commissioning issues and user error during the buildings first months of operation as was noted by a staff participant. A significantly greater number of temperature fluctuations within rooms occurred, on average, during the shoulder season.

A high percentage of rooms exhibited values below the Ministry's minimum threshold during both the heating and cooling season relative to rooms that exhibited values above the maximum threshold (which were nearly nonexistent), suggesting warmer temperatures pose greater stress on residents’ thermal comfort than colder ones and that bedroom thermostats were adjusted downwards in order to be more comfortable (Table 3).

**Table 3.** Daily Temperature Values and Trends During Cooling, Shoulder, and Heating Seasons.

Season	Daily temperature									% Time Below Threshold	% Rooms Below Threshold	% Time Above Threshold	% Rooms Above Threshold
	Min Avg. (Abs.)		Max Avg. (Abs.)		Mean		Daily Temperature Fluctuations						
	°C	°F	°C	°F	°C	°F	Min Avg (Abs)	Max Avg. (Abs.)	Mean				
Cooling	18.94 (17.94)	66.10 (64.30)	25.06 (25.61)	77.10 (78.1)	22.47	72.45	1.25 (0)	20.75 (21)	9.6	27.83	71.95	0	0
Shoulder	21.94 (21.50)	71.50 (70.70)	25.74 (26.33)	78.33 (79.4)	23.75	74.75	4.50 (2)	21.75 (23)	11.59	0.52	7.14	0.07	21
Heating	22.41 (19.00)	72.33 (66.2)	23.49 (27.52)	74.29 (81.53)	22.96	73.32	0.25 (0)	18 (19)	7.5	12.45	26.8	1.22	1.22

#### Cooling Season

During a typical cooling day, mean temperatures across rooms ranged from 18.94°C (66.10°F) to 25.06°C (77.10°F), reflecting different thermal preferences. The average of the mean temperatures calculated for each room was 22.47°C (72.45°F). Standard deviation values ranged from 0.08 to 2.96 suggesting users maintain room temperatures within a relatively narrow range. On average, each room experienced 9–10 different temperatures over a 24-hr period. Of the total number of rooms sampled, 71.95% experienced temperatures below the minimum required threshold of 22°C (71.6°F) for a given period. The total time across rooms below the minimum ministry threshold was only 27.83%, indicative of the safety measures in place. No rooms exceeded the maximum threshold of 25.9°C (78.62°F) during the sampling period.

#### Heating Season

During a typical heating day, mean temperatures across the bedrooms ranged from 22.41°C (72.33°F) to 23.49°C (74.29°F). The average of the mean temperatures calculated for each room was 22.96°C (73.32°F). Standard deviation values range from 0.06 to 2.58, indicating a relatively modest variability in temperature measurements across the different rooms. On average, each room experienced 7–8 different temperatures over a 24-hr period. Of the total number of rooms sampled, nearly a third of the rooms exhibited values below the minimum threshold. Only one room exceeded the maximum ministry threshold of 25.9°C (78.62°F) during the sampling period.

#### Shoulder Season

During a typical shoulder-season day, mean temperatures across rooms range from 21.94°C (71.50°F) to 25.74°C (78.33°F). The average of the mean temperatures calculated for each room was 23.75°C (74.75°F). Most bedrooms showed relatively stable temperature trends with minor fluctuations. Standard deviation values range from 0.15 to 1.24, indicating moderate consistency in temperature measurements across the different rooms. On average, each room experienced 11–12 different temperatures over a 24-hr period. Most bedrooms maintained temperatures well within the required thresholds. No rooms showed significant or sustained deviations from the required thresholds.

## Discussion

This article summarizes a study that identified adaptive mechanisms in LTC resident bedrooms and explored what these adaptations reveal about perceived environmental stressors. The findings are relevant in the context of creating a better understanding of the bidirectional relationships between individuals and their physical surroundings (Vischer, 2008) and suggest that adaptive comfort mechanisms in LTC resident bedrooms are both limited and highly regulated. In terms of how and how often these are used by residents, qualitative findings revealed that residents were actively using the mechanisms available to them, in this case window blinds and adjustable thermostats. Quantitative findings, however, indicated the use of these adaptive mechanisms was not shown to be an indication of a measurable IEQ inefficiency relative to the Ministry's comfort standards. Given the case study involved a new building, it was expected that the bedroom's illuminance and temperature readings would be within the required range. Despite compliance, the desire to adjust the indoor environment demonstrated a clear effort to manage or mitigate perceived stressors in their indoor environment. Some of these stressors, like privacy and heat gain, were unexpected. Residents have limited means of controlling these conditions in their environment. This study found adjustable blinds and thermostats were important attributes in supporting personal comfort. Notwithstanding their use, they also support the perception of autonomy, which literature suggests is highly important to residents (Van Hecke et al., 2019; van Loon et al., 2024; Van Steenwinkel et al., 2017). While residents spend the most time in their rooms, it is unclear, however, who was actually and regularly using the adaptive mechanism—residents, visitors, or staff. It was equally unclear how resident and room occupancy profiles impacted use. As in Tartarini et al. (2018), gaining a deeper understanding of resident activity levels, clothing, and thermal preferences would enrich the current findings. Future research could document these factors and examine how they relate to residents’ efforts to customize their bedroom environments for comfort.

*This study found adjustable blinds and thermostats were important attributes in supporting personal comfort. Notwithstanding their use, they also support the perception of autonomy, which literature suggests is highly important to residents*.

The findings presented in this article also, albeit preliminary, indicated that perceptions of physical stressors among residents may be underrepresented. It is significant to note that the control mechanisms in the bedrooms were designed to code requirements which do not have LTC residents in mind, making them inaccessible to some residents in wheelchairs or those who are bedridden. Residents with mobility issues or higher levels of physical frailty may also find it challenging to adjust their blinds. Additionally, many residents, due to physical or cognitive frailty, may not feel comfortable using or may not be able to use personal control mechanisms due to unfamiliarity with the devices or other barriers to independent use. Consequently, there could be more perceived stressors that exist and persist even when a personal control device is not used. These limitations in LTC user-building interactions complicate the understanding of comfort conditions. Given these findings, adaptations (or the lack thereof) may not accurately reflect the stress imposed by the indoor environment on residents.

*Control mechanisms in the bedrooms were designed to code requirements which do not have LTC residents in mind, making them inaccessible to some residents in wheelchairs or those who are bedridden.*

The results indicate that overall temperature distributions in resident bedrooms trend lower during both the heating and cooling seasons. While it is commonly assumed that LTC residents are more sensitive to cold than heat, this study suggests a more nuanced reality. The data, including the distribution of temperature outliers, reveals that residents may be sensitive to both hot and cold extremes. These findings highlight the need for further research into the thermoregulation challenges faced by older adults and the implications for IEQ standards in residential care settings.

Results also indicated that although light levels in resident bedrooms meet minimum regulatory requirements, they rely heavily on artificial lighting. This dependence on electric lighting not only increases energy consumption but also limits the well-established benefits of natural daylight on health and well-being. Improved daylighting strategies could reduce energy use while enhancing resident outcomes. This finding underscores a significant gap in the current LTC Home Design Manual (2015), which overlooks key aspects of resident well-being, such as sleep quality, circadian lighting, and access to daylight, that have been the focus of extensive research over the past decade. For example, Konis et al. (2018) demonstrated that increased exposure to daylight can reduce symptoms of depression in individuals with dementia living in LTC communities. The present study further suggests that resident adaptations to environmental stressors may influence daylighting conditions. Given this evidence, it is essential that minimum standards move beyond specifying lux levels—which can be met with artificial lighting—and instead mandate a minimum threshold of natural daylight to support visual comfort, circadian health, and restorative sleep.

*Although light levels in resident bedrooms meet minimum regulatory requirements, they rely heavily on artificial lighting. This dependence on electric lighting not only increases energy consumption but also limits the well-established benefits of natural daylight on health and well-being.*

Finally, the results of this study showed how occupant adaptions like the reliance on electric lighting and frequent thermostat use also become stressors on the building itself. In other words, physical environmental stressors translate into building performance stressors, compromising energy efficiency. This finding is consistent with existing literature by Laskari et al. (2022) and others on the tradeoffs that exist between comfort and performance in living environments with variable user behavior patterns.

*Occupant adaptions like the reliance on electric lighting and frequent thermostat use also become stressors on the building itself.*

### Limitations and Future Work

This study had several limitations. First, its scope was determined by the personal control mechanisms available to residents, excluding the analysis of other potential environmental stressors in resident bedrooms such as noise and indoor air quality, which also have significant health and comfort impacts in LTC settings. Second, the study focused exclusively on resident experiences, despite documented tensions in LTC between resident and staff preferences regarding IEQ.

Qualitative data collection was challenged by low participation rates, a common issue in LTC research despite inclusive recruitment strategies (Harrison et al., 2022). Additionally, the study captured data at specific points in time rather than through a longitudinal approach, limiting insights into how stressors and adaptations evolve over time.

The research was also limited to a single LTC facility, which may not reflect the diversity of building designs, operational practices, or resident populations across the sector. This limitation is compounded by the relatively recent introduction of personal control devices, such as thermostats, in LTC settings, and the lack of comparable case studies to benchmark findings. While the results are not generalizable, they provide a starting point for further research into environmental stressors, adaptive behaviors, and the role of personal control in LTC. Future studies should aim to incorporate broader variables, longitudinal data, and multistakeholder perspectives to build a more comprehensive understanding of resident–environment interactions.

## Conclusion

This study underscored the critical role of personal control mechanisms in enhancing the comfort, autonomy and well-being of residents in LTC settings. Although the building met the regulatory standards for IEQ, use of blinds and temperature changes revealed perceived or experienced stressors related to thermal and visual comfort in resident bedrooms. These findings suggest that studying adaptive behaviors can uncover important gaps in environmental design. Limitations in the accessibility and usability of personal control devices, particularly for residents with mobility or cognitive impairments, point to a significant area for improvement in LTC design. Addressing these barriers is essential to ensuring that all residents can meaningfully engage with their surroundings.

Given the limited scope of this study, its major contribution is the identification of critical opportunities for future work. In addition to those opportunities identified above, subsequent studies should examine key variables such as resident activity levels, room occupancy patterns, age, care profiles, and the actual users of control devices (e.g., residents, staff or family). These factors are integral to generating richer, more nuanced knowledge of environmental stressors and adaptive behaviors in LTC. Further investigation is also needed into the responsiveness and accuracy of newer thermostat systems, including their margin of error and impact on usage patterns. Finally, future research may investigate the integration of smart building technologies to enhance resident comfort and control. By addressing these areas, LTC design can evolve toward more responsive, adaptive, and supportive living environments.

## Implications for Practice

- The study highlights the importance of personal control mechanisms, such as window blinds and adjustable thermostats, in improving resident comfort and well-being. Designers of LTC facilities should provide these mechanisms.
- Designers of LTC facilities should consider the design of the resident bedroom and consider passive shading mechanism that ensure privacy and limit heat gain while promoting access to natural light, which can enhance visual comfort and promote better sleep quality.
- Sensor data indicated that temperature is frequently adjusted in resident bedrooms. Designers of LTC facilities should ensure that temperature control systems are easily accessible and adjustable by residents, especially those with mobility or cognitive impairments.
- Reliance on artificial lighting in resident bedrooms diminished the prospective benefits of daylight on residents’ health and well-being. Regulatory bodies should consider updating LTC standards to require appropriate daylighting.
- Future research may explore the integration of smart building technologies to enhance personal control mechanisms. These technologies can provide more responsive and adaptive environments, improving the overall quality of life for LTC residents.

## Supplemental Material

sj-docx-1-her-10.1177_19375867251406198 - Supplemental material for Environmental Stressors and Adaptive Mechanisms in Long-Term Care Resident Bedrooms: A Canadian Case Study Supplemental material, sj-docx-1-her-10.1177_19375867251406198 for Environmental Stressors and Adaptive Mechanisms in Long-Term Care Resident Bedrooms: A Canadian Case Study by Alexandra Boissonneault, PhD and Terri Peters, PhD in HERD: Health Environments Research & Design Journal
