# Supplementary material for: Environmental Stressors and Adaptive Mechanisms in Long-Term Care Resident Bedrooms: A Canadian Case Study
Source: HERD. 2026 Jan 6;19(2):150–64. doi: 10.1177/19375867251406198 (PMC12988015; doi:10.1177/19375867251406198)
Supplement: sj-docx-1-her-10.1177_19375867251406198 - Supplemental material for Environmental Stressors and Adaptive Mechanisms in Long-Term Care Resident Bedrooms: A Canadian Case Study [file sj-docx-1-her-10.1177_19375867251406198.docx]

**Semi-structured Interview Guide**
*Excerpt* (Indoor Environment)

| Primary question *(staff/residents)* | Potential prompting/probing questions |
| --- | --- |
| Can you tell me your impressions of the new building?  Can you tell me what your likes and dislikes of the new building and how these impact your experience? | 1. Where do you enjoy being most? Why? 2. Does the building pose any specific challenges to you? 3. Is there anything specifically about its design that stands out to you? 4. What are some things you like about it best? 5. What are some things you like about it least? 6. What are some things that work well? 7. What are some things that don’t work well? |
| Do you consider the indoor environment of the building to be comfortable? | 1. Do you find there is sufficient light in the building? What about the spaces you occupy most? 2. Do you find the building stuffy? Smelly? What about the spaces you occupy most? 3. Do you find the temperature in the building comfortable? What about the spaces you occupy most? 4. Do you ever have to adjust your space to make it more comfortable? 5. Do you find you have enough control when it comes to adapting your space? 6. Is there anything (else) that makes your living/working spaces uncomfortable? |

| Supplementary question *(facilities)* | Potential prompting/probing questions |
| --- | --- |
| Have building systems been performing as anticipated? | Have you encountered any technical difficulties to date?  Are there any recurring maintenance/durability/appearance issues?  Have you noticed any season-specific technical performance issues?  Is the Building Management System operating as anticipated? |
| Do you consider the user-controls to be intuitive and easy to operate? | Are there any recurring comfort issues (temperature, humidity, air flow, lighting, etc.)?  Are there any common/frequent user complains and or issues?  What have been the impacts of providing user-controlled thermostats in regularly occupies spaces? |
| Have you had to make any significant operational changes/adaptations post-handover to address recurring issues? | Do you consider the building systems easy to operate and maintain? |
